# The Evaluation of Serum Lipids Profile in Patients with Pemphigus Vulgaris: A Case-Control Study

**DOI:** 10.21315/mjms2020.27.2.7

**Published:** 2020-04-30

**Authors:** Fahimeh Rezazadeh, Maryam Moshaverinia, Farhad Handjani, Fatemeh Khoshkholgh, Nasrin Saki, Alireza Heiran

**Affiliations:** 1 Department of Oral & Maxillofacial Medicine, School of Dentistry, Shiraz University of Medical Sciences, Shiraz, Iran; 2 Molecular Dermatology Research Center, Shiraz University of Medical Sciences, Shiraz, Iran; 3 Department of Dermatology, School of Medicine, Shiraz University of Medical Sciences, Shiraz, Iran; 4 Dentistry Student, School of Dentistry, Shiraz University of Medical Sciences, Shiraz, Iran; 5 Student Research Committee, Shiraz University of Medical Sciences, Shiraz, Iran

**Keywords:** pemphigus vulgaris, cholesterol, triglycerides, serum lipids

## Abstract

**Background:**

Pemphigus vulgaris (PV) is a chronic autoimmune disease. Dyslipidemia, increased risk of atherosclerosis and higher cardiovascular morbidity, and mortality have been reported in several autoimmune conditions. It has been hypothesised that there might be an association between dyslipidemia and PV. Therefore, the objective of this study was to compare the serum lipid profile of patients with PV with healthy controls.

**Methods:**

This case-control study was carried out on 113 patients with PV and 100 healthy controls. Total cholesterol, high-density lipoprotein (HDL) and triglycerides (TG) levels were measured and low-density lipoprotein (LDL), non-HDL cholesterol (non-HDL-C) and atherogenic index of plasma (AIP) were calculated. Chi-squared test and independent Student *t*-test (or their alternatives) were used for group comparison.

**Results:**

The mean age and BMI of patients and controls were 47.7 ± 14.5 and 28 ± 6.2 and, 44.5 ± 18.5 and 25.5 ± 5.1, respectively. Total cholesterol, LDL, HDL, non-HDL-C and TG were statistically different between the two groups (*P* values < 0.001; < 0.001; < 0.001; < 0.001 and 0.021, respectively). However, AIP was not significantly different (*P-*value = 0.752).

**Conclusion:**

The serum lipid profile was significantly higher in PV patients compared to healthy controls. Therefore, PV patients may be more prone to develop atherosclerosis and this finding can be important in the overall management of these patients.

## Introduction

Pemphigus vulgaris (PV), a sub-type of pemphigus, is a bullous skin disease involving the skin and mucosa. This autoimmune blistering disease is characterised by Immunoglobulin G (IgG) autoantibodies targeted against cell adhesion molecules, desmogleins (DSG) −1 and −3 (1–6). DSG-1 is expressed throughout the epidermis and is highly concentrated in the superficial layers, whereas DSG-3 is expressed in the parabasal and basal layers. In the mucosa, both DSG-1 and DSG-3 are expressed throughout the epidermal layers (5, 7–10). In addition, it has been suggested that endoplasmic reticulum (ER) stress is associated with disease pathogenesis (11). The global incidence of PV is approximately 0.076–5/100,000 and women are more prone (1:1.1–2.25 male to female ratio) (5, 12–13). In Iran, the incidence of PV and male to female ratio are 5/100,000 and 1:1.6, respectively (14).

Altered serum lipid levels were investigated in several autoimmune diseases such as rheumatoid arthritis and systemic lupus erythematosus (SLE) (15–16). This suggests that immune response might be associated with atherogenesis, the major cause of cardiovascular diseases (17).

Recently, several studies have proposed the role of increased ER stress and oxidative stress in PV pathogenesis and (or) progression (11, 18–19). On the other hand, these two processes are also linked to hyperlipidemia and atherosclerosis (20–21). Hence, theoretically, it appears that there might be an association between dyslipidemia and PV. In this study, we compared the serum lipid profile of patients with PV with healthy controls.

## Methods

In this case-control study, 150 patients with biopsy proven PV using immunofluorescence (IF) and histopathology findings, who were admitted to the dermatology ward of Faghihi Hospital, affiliated with Shiraz University of Medical Sciences from 2012–2015, were enrolled. Exclusion criteria included patients with dyslipidemia, fatty liver, diabetes mellitus, metabolic syndrome, history of stroke, hypertension, cardiovascular diseases, obstructive liver disease, renal diseases, connective tissue diseases, other subtypes of pemphigus, history of smoking and alcohol consumption, family history of dyslipidemia, or the use of drugs interacting with serum lipid measures (cyclosporine, corticosteroids, β-blockers, thiazides, retinoids, methotrexate and statins, and etc.). Accordingly, 37 patients were excluded based on the above criteria and assessments were done on the remaining 113 patients. One hundred healthy controls, who did not meet the exclusion criteria, were selected through convenience sampling from the emergency ward of the same hospital. Matched case-control methodology was not applied.

The demographic variables (age, gender and body mass index [BMI]) were recorded. After fasting for 14 h, a 5 mL venous blood sample was taken by a sterile syringe from all participants in the morning and was sent to the hospital laboratory for further analysis. Serum total cholesterol, triglycerides (TG), low-density lipoprotein (LDL) and high-density lipoprotein (HDL) were measured by the enzymatic method using a standard kit (Bionik Diagnostic Systems, Iran). Also, non-HDL cholesterol (non-HDL-C), total cholesterol minus HDL and atherogenic index of plasma (AIP) (log [TG_mmol l_/HDL_mmol l_ ]) were calculated. AIP is a novel metrics in dyslipidemia management, which is a better marker in predicting coronary artery diseases in comparison to single lipid measures such as LDL. We used the Adult Treatment Panel III (ATP III) guidelines from the USA’s National Cholesterol Education Programme for normal ranges of the measured parameters.

Rattle GUI (graphical user interface) package powered by R programming language (version 3.3.1 for Windows) was used for statistical analysis. The univariate analysis was done using Chi-squared test (or Fisher’s exact test) or independent Student *t*-test (or Wilcoxon rank-sum test) in order to evaluate the difference between the two groups. *P*-value < 0.05 was considered to be statistically significant.

## Results

One hundred thirteen cases (50 males and 63 females) were compared with 100 healthy controls (59 males and 41 females). No missing value was in the dataset. The mean age in the case and control group were 47.7 ± 14.5 years and 44.5 ± 18.5 years, respectively. Both groups were overweight, as the mean BMI was 28 ± 6.2 and 25.5 ± 5.1 in the case and control group, respectively. Regarding baseline demographic variables, no statistical difference was observed for age (*P* value = 0.093), but groups were different according to gender (*P-*value = 0.034) and BMI (*P-*value = 0.001) (Table 1).

Table 2 depicts the case-control lipid profile. In the case group, the mean total cholesterol, LDL, HDL, TG, non-HDL-C and AIP were 190.4 ± 43.3 mg/dL, 115.9 ± 36.5 mg/dL, 46.6 ± 11.5 mg/dL, 135.6 ± 73 mg/dL, 143.8 ± 39.5 mg/dL and 0.0667 ± 0.201, respectively. In the control group, these measures were 155.25 ± 37.3 mg/dL, 92 ± 33.3 mg/dL, 40.2 ± 13.1 mg/dL, 115.5 ± 52.7 mg/dL, 115 ± 33 mg/dL and 0.077 ± 0.265, respectively.

The LDL and non HDL-C levels were above the upper limit among the patients. Univariate analysis showed that total cholesterol, LDL, HDL, non-HDL-C and TG were significantly higher in the patient group (*P-*values < 0.001; < 0.001; < 0.001; < 0.001 and 0.021, respectively). However, AIP was not significantly different between the two groups (*P-*value = 0.752).

## Discussion

To our knowledge, Wohl et al. (22) were the first to carry out such a study on the serum lipids profile in PV. In line with our results, they reported that elevated total cholesterol and TG are associated with PV. This finding was confirmed even after controlling for confounding factors. Considering the scarce literature on this subject, only several studies on other skin autoimmune diseases were found. In a study by da Cunha et al. (23), pemphigus foliaceus was linked to a higher serum TG level. Among patients with oral lichen planus, higher Castelli’s atherogenic index, TG, total cholesterol and, LDL and lower HDL levels were reported (24, 25). In another study by Taheri et al. (26), patients with psoriasis had a higher plasma lipid profile. In addition, altered serum lipid profile was observed in rheumatoid arthritis, SLE, antiphospholipid syndrome and systemic sclerosis (15–16). Notably, these studies, like ours, were not performed with large sample sizes and BMI, age and gender matched control groups, which could all lead to bias. Change in serum lipid profile in such patients implies that the immune response might be involved in atherogenesis (17). Additionally, the pattern of dyslipidemia differs among various autoimmune diseases, but they all may share the same atherogenic mechanisms (27).

One mechanism that might explain the relationship between dyslipidemia, atherogenesis and autoimmunity is the lipid peroxidation of LDL, which is the key event in the initiation and progression of atherosclerosis. Oxidised low-density lipoprotein (ox-LDL) promotes endothelial dysfunction and pro-inflammatory cytokine release, leading to an autoimmune response that accelerates the intracellular accumulation of lipids within atherosclerotic plaques (28–29) by macrophage scavenger receptors (30). This ox-LDL induces anti-ox-LDL-antibody production which is specific for autoimmune disorders (31–33). Also, in both autoimmune and non-autoimmune atherosclerosis, ox-LDL binds to β2-glycoprotein I (β2GPI) which forms a circulating complex (ox-LDL/β2GPI complex). It is likely that β2GPI and/or ox-LDL/β2GPI complex contributes to early atherogenesis by stimulating pro-inflammatory innate immunity through endogenous sensors and inflammasome/interleukin-1 pathways (29, 34).

It is suggested that oxidative stress and ER stress are two pivotal processes in hyperlipidemia and atherosclerosis development (20–21). In addition, recently published studies (11, 18–19) have found the association between these two mechanisms and PV pathogenesis and (or) progression; however, whether increased oxidative stress causes disease manifestations and/or activity or vice versa still remains unknown. The development of ER stress is linked to PV progression (35–36). The protein kinase RNA-like ER kinase (PERK) activates the pro-apoptotic transcription factor that is an enhancer-binding protein homologous protein (CHOP), which induces ER stress-associated cell death (37–39). Furthermore, it is likely that cell exposure to anti-DSG-1 antibodies partially simulates the underlying pathogenic mechanism. The anti-DSG-1 antibodies cause acantholysis, which is the upper layer detachment from the basal membrane, which decreases nutrient supply, homeostasis and normal cell growth, and specifically induces ER stress (20, 40).

The present study showed a higher serum LDL level in PV patients. Non-HDL-C is probably the best predictor among all cholesterol measurements both for coronary artery events and strokes (41), and this was also higher among PV patients. Both LDL and non-HDL-C measures were above the normal range. AIP is a marker of lipoprotein particle size, which adds an effective value beyond single lipid measures to predict the risk of atherosclerosis and coronary artery diseases (42–47); however, this marker was not significantly different between PV patients and the control group. One possible explanation might be the fact that the best predictability performance of AIP is when a patient has other cardiovascular risk factors (48).

## Conclusion

In summary, serum lipid profile was statistically different between PV patients and healthy controls; hence, PV patients might be more prone to develop atherosclerosis. Further studies in different populations, with larger sample sizes and considering more variables like severity and duration of the disease, as well as more reliable designs, like cohort studies, are warranted to further validate our findings.

## Figures and Tables

**Table 1:** Demographic characteristics of the patients

	PV (*n* = 113)	Control (*n* = 100)	*P-*value 2
Female	63 (55.75%)	41 (41%)	0.034
Age, mean ± SD^1^, year	47.7 ± 14.5	44.5 ± 18.5	0.093
BMI, mean ± SD, kg/m^2^	28 ± 6.2	25.5 ± 5.1	0.001

Note:

1 standard deviation;

2 *P-*value < 0.05 = statistically significant difference

**Table 2:** Serum lipids profile in the case and control groups

Lipid or lipoprotein	PV (*n* = 113)	Control (*n* = 100)	*P-*value^1^
Total cholesterol, mg/dL (< 200)	190.4 ± 43.3	155.25 ± 37.3	< .001^2^
LDL, mg/dL (< 100)	115.9 ± 36.5	92 ± 33.3	< .001^2^
HDL, mg/dL (40–60)	46.6 ± 11.5	40.2 ± 13.1	< .001^2^
TG, mg/dL (< 150)	135.6 ± 73	115.5 ± 52.7	0.021^3^
non-HDL-C, mg/dL (< 130)	143.8 ± 39.5	115 ± 33	< .001^2^
AIP (low risk < 0.1)	0.067 ± .201	0.077 ± .265	0.752^3^

Notes:

1 *P-*value < 0.05 = statistically significant difference;

2 Wilcoxon rank-sum test;

3 independent Student *t*-test; normal range based on ATP III guidelines
